# Nano and Microparticles as Potential Oral Vaccine Carriers and Adjuvants Against Infectious Diseases

**DOI:** 10.3389/fphar.2021.682286

**Published:** 2021-06-02

**Authors:** Seyed Davoud Jazayeri, Hui Xuan Lim, Kamyar Shameli, Swee Keong Yeap, Chit Laa Poh

**Affiliations:** ^1^ Centre for Virus and Vaccine Research, Subang Jaya, Malaysia; ^2^ Malaysia-Japan International Institute of Technology, Universiti Teknologi Malaysia, Kuala Lumpur, Malaysia; ^3^ Department of Marine Biotechnology, China-Asean College of Marine Sciences, Xiamen University Malaysia, Sepang, Malaysia

**Keywords:** nanoparticles, microparticles, infectious diseases, oral vaccines, vaccine carriers

## Abstract

Mucosal surfaces are the first site of infection for most infectious diseases and oral vaccination can provide protection as the first line of defense. Unlike systemic administration, oral immunization can stimulate cellular and humoral immune responses at both systemic and mucosal levels to induce broad-spectrum and long-lasting immunity. Therefore, to design a successful vaccine, it is essential to stimulate the mucosal as well as systemic immune responses. Successful oral vaccines need to overcome the harsh gastrointestinal environment such as the extremely low pH, proteolytic enzymes, bile salts as well as low permeability and the low immunogenicity of vaccines. In recent years, several delivery systems and adjuvants have been developed for improving oral vaccine delivery and immunogenicity. Formulation of vaccines with nanoparticles and microparticles have been shown to improve antigen stability, availability and adjuvanticity as well as immunostimulatory capacity, target delivery and specific release. This review discusses how nanoparticles (NPs) and microparticles (MPs) as oral carriers with adjuvant characteristics can be beneficial in oral vaccine development.

## Introduction

Mucosal immunization has numerous advantages over parenteral (needle-based) administrations such as socio-economic benefits, relatively improved safety by a lower risk of needle injury infection/inflammation, self-delivery, capacity for mass immunizations, no special training required, patient compliances and ability to elicit mucosal immune responses (Corthésy and Bioley, 2018). Mucosal membranes are exposed to antigenic substances which can induce specific antibody as well as cell-mediated immune responses through the secretory IgA (sIgA) that could prevent the attachment of bacteria and viruses to the mucosa and plays a major role in mucosal protection. The IgA following oral vaccination could migrate to distant mucosal sites, like respiratory and urinogenital mucosa, by forming a defense network against bacterial and viral pathogens (Pietrzak et al., 2020).

Intranasal and oral routes are both considered as the main options for mucosal immunization. As intranasal immunization could have some harmful effects on people with asthma and other chronic pulmonary or cardiovascular disorders, the oral route appears to be the safest, more applicable and most preferred mode of vaccination for the development of new generation vaccines.

The gastrointestinal (GI) tract of humans has over 260–300 m^2^ of the mucosal surface containing immune inductive tissues such as Peyer’s patches (in the small intestine), lymphoid follicles (large intestine) and intraepithelial lymphocytes which are important for antigen presentation and appropriate functioning of the immune system Zhang et al. (2018).

Currently, a limited number of oral vaccines that have been licensed for human use are using live attenuated (Polio; OPV, Typhoid; Vivotif, Cholera; Orochol, Rotavirus; Rotarix and RotaTeq) or whole inactivated pathogens (Cholera; Dukoral) (Mwanza-Lisulo and Kelly, 2015). The commercially available oral vaccines have shown high efficacy in industrialized countries but much lower efficacy in low or middle-income countries (Lestari et al., 2020). Although the impaired efficacy is not very well understood, some possibilities for the lower efficacy included nutritional factors such as vitamin A, interaction with the high titers of antibody in maternal breast milk, environmental enteropathy (Qadri et al., 2013) and *Helicobacter pylori* infection (Muhsen et al., 2014).

On the other hand, oral vaccine (peptide, DNA, or RNA-based) delivery has always been a significant challenge for pharmaceutical technology and vaccine development due to its very poor bioavailability through the GI tract. The possible explanation for the low oral absorption of the vaccines might include enzymatic degradation, poor membrane penetration, hepatic metabolism and the unique physicochemical characteristics of the GI mucosa (Xu et al., 2018).

As the GI tract is continuously exposed to a broad range of pathogens, successful oral vaccines need to induce appropriate strong signals to be recognized by the immune system, otherwise, the host immune system would consider the vaccines as non-immunogenic and resulting in immune tolerance instead of conferring broad protection. Therefore, it is important to design an effective vaccine carrier including safe effective adjuvants to sufficiently stimulate the mucosal immune system (Vela Ramirez et al., 2017). An ideal oral vaccine carrier is expected to protect the antigens from degradation through the GI tract, deliver sufficient antigens to the inductive mucosal surface, enhance antigen uptake, activate immune cells, produce effective long-lasting mucosal and systemic immune responses. During recent years, various strategies have been developed for effective oral vaccine delivery, such as enzyme inhibitors, encapsulation into particulate delivery systems, chemical modifications, preparation of macromolecular conjugation and targeted delivery to the colon. Nevertheless, based on currently available data none of these approaches could be considered as a breakthrough.

Most of the soluble antigens are unable to be efficiently endocytosed by the antigen-presenting cells (APCs) and cannot induce any protective immunity. Conjugation or encapsulation of soluble antigens with nanocarriers could improve their immunogenicity and facilitate recognition and uptake of antigens by APCs. Therefore, the immunogenicity of soluble antigens could be improved by conjugation or encapsulation with nanocarriers that could facilitate the recognition and uptake by APCs (Pati et al., 2018). Several oral vaccine formulations are currently being explored based on nanoparticles (NPs) and microparticles (MPs). NPs and MPs can be administered via subcutaneous, intramuscular or through mucosal sites (oral and intranasal routes) as well as penetrating capillaries. Incorporation of antigens in NPs and MPs could be achieved by physical encapsulation or by covalent conjugation (Chattopadhyay et al., 2017). Encapsulation could protect the structure of antigens against proteolytic degradation, improve immunostimulatory effects and antigen delivery to APCs. Due to poor immunogenicity of recombinant and synthetic antigens in different vaccine platforms, an adjuvant in vaccine formulation could increase immunogenicity, reduce the amount of antigens, improve the immune responses and protection (Wang and Xu, 2020). This review focuses on the potential applications of various types of NP and MP systems as novel delivery and enhancement of adjuvanticity for oral vaccine development against infectious diseases.

## The Microfold-Cells

The surface of the GI tract contains a chemical and physical barrier formed by an impermeable layer of epithelial cells. The M cells are mainly located within the epithelium of Peyer's patches in the ileum and have some noticeable features for the uptake of particles. However, the lower percentage of M cells in the GI tract (1% of the total surface of the intestine) causes significant problems in humans towards oral vaccine development. The efficacy of oral vaccination is mostly impaired due to the low populations of M cells in the intestines. To increase the efficiency of the delivery of oral vaccines, it is necessary to target the vaccine complex to M cells. Various absorption parameters that play an important role in the uptake of NPs/MPs via M cells include particle size, charge, hydrophobicity/hydrophilicity balance and existence of a targeting molecule at the surface of the particles. Generally, M cells could uptake particles ranging from below 1 µ to above 5 µ in size. Particles smaller than 1 µ are passed into the basal medium, while particles above 5 µ are delivered to Peyer’s patches. The optimum size of NPs for transcytosis by M cells is proposed to be smaller than 200 nm. The formulation of negatively charged and hydrophobic particles is favorable because of the optimal absorption by M cells (Des Rieux et al., 2006).

The intestinal immune system is regulated by gut-associated lymphoid tissues (GALT) which contain inductive and effector sites. Inductive tissues include the Peyer’s patches, lymphoid follicles (within lymph nodes), and APCs. Meanwhile, effector sites comprise the lamina propria and the surface epithelium. After oral administration with particulate vaccines, antigens could migrate through the GI tract.

After entering the small intestine, specialized M cells in the Peyer’s patches sample and transport the antigens across to APCs. The antigens are processed into small fragments by DCs that present antigenic fragments on their surface.

These antigen-loaded DCs provide costimulatory signals to activate naive CD4 T cells. The primed helper T cells further interact with antigen-specific B-cells that undergo class-switching to become immunoglobulin-secreting cells.

**FIGURE 1:**
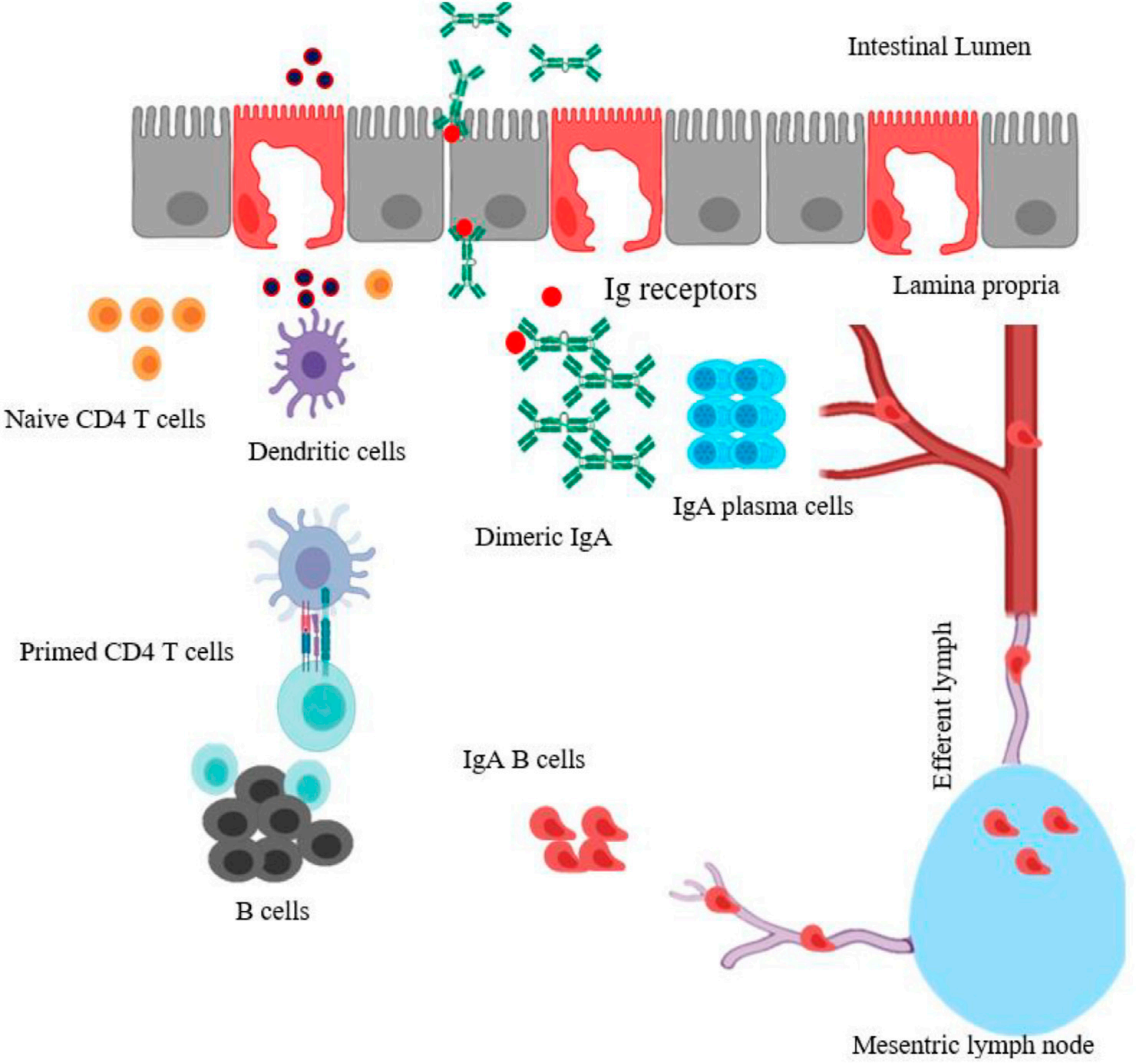
Schematic diagram of mucosal immune induction to generate T-cell-dependent IgA production. The particulate antigens in intestinal lumen are moved across the intestinal epithelium barrier by sampling M cells, transcytosed and presented to DCs. The antigen-loaded DCs (activated DCs) could travel and prime naïve CD4 T cells in Peyer’s patches. Primed CD4 T cells then activate B cells, which undergo isotype switching, thus generating antigen-specific IgA^+^ B cells. These IgA^+^ B cells leave the Peyer’s patches through the afferent lymph system to mesenteric lymph node, enter the blood circulation and reach effector sites in the lamina propria, mature, and become IgA producing-plasma B cells. The dimeric or polymeric IgA binds to Ig receptors expressed on the basolateral surface of epithelial cells to form SIgA. The figure is made with biorender (https://biorender.com/).

Upon maturation, IgA B cells leave the Peyer’s patches through afferent lymphatics to the regional mesenteric lymph node before reaching the systemic blood circulation (Figure 1) (Brayden et al., 2005; Marasini et al., 2014; Vela Ramirez et al., 2017). Finally, the circulating antigen-specific IgA secreting B cells migrate to distant effector sites in the lamina propria undergo differentiation and maturation to generate high-affinity IgA producing plasma cells which produce the dimeric or polymeric form of IgA. The dimeric or polymeric IgA binds to polymeric Ig receptors expressed on the basolateral surface of epithelial cells to form SIgA which further translocates toward the luminal surface of the intestine (Marasini et al., 2014).

## Adjuvants

Adjuvants originated from the Latin word “adjuvare”, which means “aid or to help” and was first explained by Ramon in 1924 as “substances used in combination with a specific antigen to enhance the immunological responses” (Awate et al., 2013).

Generally, adjuvants are non-specific immunopotentiators which together with the antigen(s), could boost the body's immune responses as well as changing the type of the immune response (Jin et al., 2019).

Aluminum salts, developed in the United States in the 1920s and for more than 7 years, was the only adjuvant available in the United States until MF59 (incorporated in influenza vaccine) was approved in the 1990s (Del Giudice et al., 2018).

Alum is still an important component of most licensed human vaccines like human papillomavirus (HPV), Hepatitis A virus (HAV), diphtheria, Hepatitis B virus (HBV), *Haemophilus*, Influenzae Type b (Hib), tetanus, and meningococcal vaccines (Lee and Nguyen, 2015).

Alum as a potential adjuvant have been tested in the formulations of a few under exploratory and pre-clinical coronavirus vaccine investigations. Liang et al. (2020) demonstrated formulation of alum with S protein or receptor-binding domain (RBD) which significantly improved the titers of IgG1 in serum, increased high affinity of neutralizing antibodies as well as generated long-lasting memory B cells in mice (Liang et al., 2020).

Furthermore, virus-like particle (VLP) and inactivated vaccines containing E, M, and N proteins were formulated with Alum and showed enhanced IgG1 and neutralizing antibody titers and prolonged durability. Studies also demonstrated that alum adjuvant plays an essential role in the dose-sparing of CoV vaccines (Liang et al., 2020). Moreover, recently Gao et al. (2020) showed that a purified inactivated SARS-CoV-2 vaccine in combination with aluminum hydroxide as adjuvant could provide complete protection in rhesus macaques with potent humoral responses and with no lung immunopathology (Gao et al., 2020).

Aluminum-based adjuvant might be sufficient for eliciting humoral immune responses with acceptable safety and efficiency, but it is a poor immunostimulator of cellular immune responses and has limited application as an adjuvant for vaccination against intracellular pathogens. Aluminum is not effective at triggering the molecular events that support IgA class switching, recombination or homing of activated T and B cells in mucosal tissues (Reed et al., 2009). Therefore, new generation of adjuvants for improvement of the immunogenicity of weak antigens (Ags), with limited or no toxicity and side effects, effective with low-dose Ags, suitable with many different Ags, effective enough to reduce the number of immunisations, simultaneous stimulations of humoral, cellular and mucosal as well as long-term immune stimulations and responses are required.

Adjuvant selection and formulation can be based on several parameters including the type of disease, route of vaccination, vaccine platform, physical and chemical natures of antigen, type of required immune response and age of the target population. Moreover, the selection of the wrong adjuvant could reduce vaccine efficiency. Thus, the selection of vaccine antigens must take into account the selection of adjuvant to enhance the potential effectiveness of vaccine candidates (Reed et al., 2013).

**FIGURE 2:**
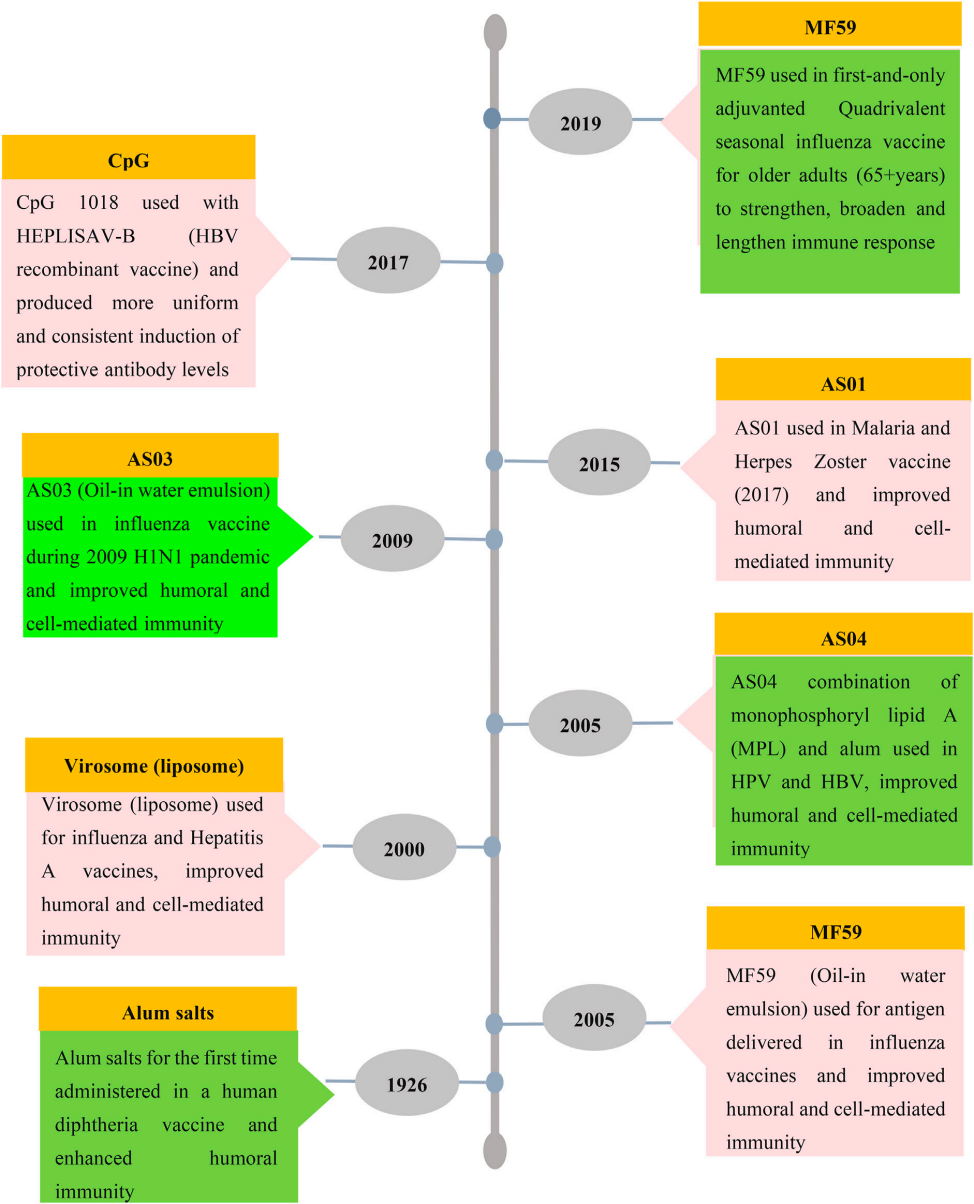
Timeline of licensed vaccine adjuvants. Aluminium salt was the first and most used adjuvant with other limited adjuvants such as MF59, virosome, AS01, AS03, AS04 and CpG ODN which are used in FDA-approved vaccines for humans.

Many different types of compounds have been evaluated as adjuvants for human and animal applications and these include; mineral salts, microbial products, emulsions, saponins, cytokines, polymers, NPs, MPs and liposomes. To date, only a few adjuvants have been used in licensed human vaccines: including Alum (Lee and Nguyen, 2015), MF59 (composed of squalene droplets stabilized with surfactants Tween 80 and Span 85) (Ko and Kang, 2018), squalene-based adjuvant AS03 (Wilkins et al., 2017), AS04 (monophosphoryl lipid A (MPL) + alum) (Wang and Xu, 2020), AF03 (squalene-based emulsion adjuvant) (Wang and Xu, 2020), virosomes and heat-labile enterotoxin (LT) (Tregoning et al., 2018). Figure 2 shows the timeline of the development of licensed vaccine adjuvants for humans. Another application of adjuvants comprises delivery and stabilizing antigens, which could promote more effective delivery of immunogens and at the same time enhance antigen-specific immune responses.

### Nanoparticle-Based Delivery System for Mucosal Vaccine

Even though adjuvants derived from bacteria or plant components and synthetic materials are immunostimulatory, many of them exhibited toxicity, leading to undesirable adverse reactions (Sander et al., 2019). These adjuvants also lacked the ability to overcome mucosal barriers and deliver the antigens to mucosal APCs or M cells for processing. NPs are good delivery systems as they are capable of traversing mucosal barriers to efficiently target the immune cells, control the duration release and presentation of antigens. Antigens can either be encapsulated or surface absorbed or chemically conjugated to the NPs. NPs represent a promising platform that combines both delivery and immunostimulatory functions. They have also demonstrated safety, efficacy and ability to stimulate mucosal and systemic immune responses (Jazayeri and Poh, 2019).

### Nanoparticles and Microparticles

NPs technically range in size from 1 to 1,000 nm (1 μm) (Jeevanandam et al., 2018) while MPs are particles with sizes ranging from 1 to 1,000 μm (Lengyel et al., 2019). The size of the particulate immunogens has different effects on oral distribution, targeting ability and types of induced immune responses. Small particles are more efficient in permeating biological barriers, moving through capillaries and distribute in blood circulation. NPs with a diameter of 100 nm or less are preferred over larger particles for targeting drug delivery purposes. Moreover, the size of particles could affect cellular specificity and migration. Additionally, small NPs ranging from 20 to 200 nm could rapidly drain to the lymph nodes (LN), where they could be taken up by resident DCs. Large NPs from 500 to 1,000 nm are dependent on cellular transport by DCs, migrating from the injection site (skin) to LN *in vivo*. These data suggested that larger NPs prefer interacting with tissue-resident APCs, while smaller NPs (<200 nm) could circulate through the vein and lymphatic drainage, thus providing better antigen presentation (Liu et al., 2017). Moreover, MPs promote humoral immune responses whereas NPs tend to favor the induction of cellular immune responses (Silva et al., 2016). The NPs taken up into M cells in Peyer’s patches are efficiently transferred to dendritic cells which can initiate an immune response (Cao et al., 2019).

## Lipid-Based Vehicles

### Liposomes

Liposome technology was established by Bangham and Horne in the 1960s as a system for the diffusion of ions across biological membranes and later in the 1970s, there was an interest in drug delivery applications (Bernasconi et al., 2016). Liposomes could mimic the natural structure of cell membranes and have long been investigated as drug carriers due to excellent entrapment capacity, safety and biocompatibility (He et al., 2019). Liposomes are spherical vesicles characterized by a bilayer of phospholipids with an internal aqueous cavity. The injectable formulations of liposomes have been used as delivery vehicles and licensed for clinical use (Bulbake et al., 2017).

The cationic liposomes have been extensively considered as an effective oral vaccine carrier for diverse antigens and adjuvanticity applications due to their unique properties such as biodegradability, biocompatibility, surface charge variability, and membrane fluidity (Table 1). Encapsulation of water-soluble molecules such as proteins, peptides, nucleic acids, haptens, or carbohydrates are in the aqueous inner layer while lipophilic compounds such as lipopeptides, antigens, adjuvants or linker molecules can be included in the external section of liposomes (Schwendener, 2014). Liposomes could protect the payload from the harsh GI environment, control the release of active drugs and induced both humoral and cell-mediated immune responses (Vela Ramirez et al., 2017).

**TABLE 1:** Advantages and disadvantages of the different types of nanocarriers.

Nanoparticle system	Advantages	Disadvantages
Liposomes	- Biodegradable, biocompatible, non-toxic and non-immunogenic	- The formulation is highly dependent on charge and size of the antigen
	- Safety due to the resemblance to biomembranes	- Instability and poor permeability
	- Protect encapsulated hydrophilic, hydrophobic and amphipathic antigens	- Require special storage
	- Can be formulated to NPs or MPs and administered through various routes	- Encapsulated antigens fail to reach M cells and release/degradation in GI
	- Protect the immunogen through GI tract, improve transfection and controlled release	- Low solubility
		- Short half-life
		- High cost
Bilosomes	- Self-adjuvant properties	- Unstable in the GI environment
	- Do not require special storage	
	- High antigen encapsulation	
	- Protect antigens in GI tract, rapid and efficient uptake by M cells	
	- Induce mucosal immunity at the site and other distant mucosal sites	
PLGA	- Biodegradable, biocompatible, and non-toxic	- Instability of antigens during encapsulation, drying and storage
	- Can be formulated to NPs or MPs	
	- Various antigens with full antigenicity can be loaded within PLGA- or PLGA-based conveyor	
	- Can be recognized by professional APCs	
	- Approved by the US food and drug administration	
ISCOM	- Small amounts of encapsulated antigens are immunogenic	- Incorporation of many antigens into the structure is difficult
	- Induce humoral and cellular immune responses	- Not very stable in the gut
	Highly stable	- Difficult to manufacture
		- Strong pain at the injection site
		- Strong toxic reactions
Gold NPs	- Readily internalized by macrophages and dendritic cells	- Accumulate in organs such as liver and spleen for long periods which could ultimately be associated with toxicity
	- A wide range of molecules, (adjuvants and antigens) can be conjugated	
	- Large scale production is possible	
Chitosan	- Non-toxic, biodegradable, biocompatible, and has bio-adhesion ability	- Insoluble at physiological pH in water
		- Easy degradation in acidic media such as the GI tract
		- Irregular distributions
Alginate	- Low toxicity, biocompatibility, biodegradability	- Incompatible with heavy metals
	- Approved by the U.S. Food and drug administration	- Cannot be fully eliminated from our body
	- Stable in gastric fluid	- Non-degradable in mammals

Liposomes significantly protect the delivery of DNA vaccines in the GI against DNases and promote absorption at the cellular level. The fusion of liposomes to host cells could facilitate the internalization of encapsulated DNA vaccine into the cytoplasm and adjuvanticity of liposomes could enhance the expression of the recombinant plasmid (Schwendener, 2014).

Wang et al. (2010) constructed a DNA vaccine encoding *Mycobacterium* antigen 85A (Ag85A) encapsulated in liposomes. After three oral vaccinations in mice, the Ag85A protein antigen was detected in the epithelium, M cells, dendritic cells (DCs) and Peyer’s patches of the small intestine and antigen-specific mucosal, systemic humoral and cellular immunity against *tuberculosis* were generated (Wang et al., 2010).

Biopolymer-coated liposomes as a potential carrier was able to successfully deliver DNA/GFP through the Peyer’s patches of mice, which are mostly located in the ileum, and extended the stability of surface charge of the particles which were crucially important for oral DNA vaccine delivery. The biopolymer such as chitosan as a biodegradable coating material with liposomes was found to improve DNA internalization efficiency, reduced DNA deterioration, increased positive surface charges and facilitated DNA encapsulation (Channarong et al., 2011). Biopolymers bonded on the surface of liposomes could change their surface charge, for example, their surface charge would be positive in the presence of chitosan. The cationic liposomes were used for oral delivery of a DNA vaccine encoding the M1 gene of influenza A virus. Oral immunizations of mice three times at weekly intervals showed there was expression of the M1 gene in the intestine, elicited both humoral and cellular immune responses as well as protection against respiratory challenge. These findings suggested cationic liposomes in the GI could be considered as a potential carrier and route for DNA vaccine delivery against viral infections (Liu et al., 2014).

A variety of liposome-based vaccines as oral carriers were administered to target a wide range of viral and bacterial diseases in veterinary medicine. Oral vaccination of chickens with a liposome-associated carrier with the recombinant SefA (rSefA) protein from *Salmonella enteritidis* generated both systemic and mucosal antibody responses. A significant reduction of intestinal bacterial colonization in the Gl tract was observed after an oral challenge with live *Salmonella enteriditis* (Pang et al., 2013). Oral immunization of chickens with commercial Newcastle disease (ND) vaccine (LaSota strain) and combination of gypenosides (GPS)/liposome as adjuvant significantly enhanced lymphocyte proliferations, increased specific antibody titer against ND and promoted cytokine secretion. Therefore, the results indicated formulations of GPS and liposome could further enhance the immune response against ND vaccine compared with GPS or liposomes alone (Yu et al., 2013). In another study, live ND vaccine (LaSota strain) encapsulated in 1,2-dioleoyl-3-trimethylammonium propane (DOTAP)-based liposomes was used. Chickens orally vaccinated with the liposomal ND vaccine showed higher antibody titer and indicated encapsulation of ND vaccine in DOTAP-based liposome induced significantly higher immunity than the live ND vaccine (Onuigbo et al., 2012).

*H. pylori* urease B subunit and cholera toxin B were expressed and purified in *E.coli* as a recombinant fusion peptide. The recombinant fusion peptide was encapsulated in the phospholipid bilayer vesicle liposome with encapsulation efficiency of 71.4%. Liposome as the oral carrier could protect fusion peptide in the GI tract from protease, acids and other harmful components. Oral vaccinations of mice with liposome-encapsulated recombinant fusion peptide administered at four weekly intervals were able to increase the specific IgG and IgA antibodies as well as providing prophylactic and therapeutic effects against *H. pylori* infection (Zhao et al., 2007). In another study, Dhakal et al. (2018) used liposome NPs as a carrier to incorporate ten highly conserved T and B cell epitope peptides. Intranasal mist immunization of pigs with liposomal conserved peptide vaccines was found to enhance the frequency of peptide specific cellular (virus-specific T-helper/memory cells) and mucosal humoral immune responses (Dhakal et al., 2018).

Released fractions of labile biomacromolecules are degraded quickly and will not be absorbed through GI and only liposomes that could survive in the GI environment and manage to penetrate the mucus layers could reach the intestinal epithelia and be absorbed together with the payloads. The initial challenge to enhance the oral absorption of liposomes as well as the payloads was to maintain the integrity of liposomes and prolong gastrointestinal residence, thus enhancing penetration of the mucus layers. Recent advances are focused on modulating the compositions of the lipid bilayers or modifying the liposomal surfaces with polymers or ligands to modulate the *in vivo* fate of liposomes after oral administration (He et al., 2019).

## Nanostructured Lipid Carrier

Nanostructured Lipid Carrier (NLC) is a type of lipid NPs containing a hydrophobic solid and liquid lipid core covered by the surfactant monolayer (Gómez-Aguado et al., 2020). Compare to other lipid systems, NLC possessed the advantages of low toxicity, simple production, can be subjected to sterilization and is more affordable (Beloqui et al., 2017). NLCs were primarily considered for the delivery of lipophilic drugs but their suitability for hydrophilic drugs is now well established (Salvi and Pawar, 2019).

NLC has been recognized as a nano-delivery solution to increase the oral bioavailability of poorly soluble drugs (Beloqui et al., 2014). Interestingly, recent studies have reported the potential of using NLC to deliver replicating viral RNA (rvRNA) (Erasmus et al., 2018) and HIV p24 peptide vaccines (Bayon et al., 2018) to achieve protection in the *in vivo* studies. However, both studies delivered the NLC-rvRNA and HIV p24 peptide vaccines through intramuscular (IM) and intraperitoneal injections (IP), respectively. Thus, further studies to evaluate the potential or even to improve the formulation of the NLC-mRNA or NLC-peptide vaccines as oral vaccines can be considered.

### Bilosomes

In addition to traditional liposomes, bilosomes are non-ionic lipid-based vesicles (niosomes) containing biodegradable and biocompatible bile salts (sodium deoxycholate). They have been extensively used for oral vaccine delivery due to their adjuvanting properties, encapsulation of antigens, flexible size formation, and rapid uptake by M cells (Table 1). Recently, several studies have been performed using the bilosomes as a carrier for oral vaccine delivery and was reported to efficiently induce mucosal sIgA immunity at local and other distant mucosal sites.

As most of the hepatitis B vaccines were administered via the parenteral route and they failed to provide mucosal immune responses, oral vaccination could be considered as an alternative to overcome this failure. Oral immunization of mice with bilosomes containing the recombinant hepatitis B surface antigen was found to increase uptake of bilosomes entrapped in gut-associated lymphoid tissues and produced both systemic as well as mucosal antibody responses (Shukla et al., 2008).

Jain et al. (2005) showed mannosylated bilosomes encapsulating DNA vaccine encoding the hepatitis B antigen could be used as an oral carrier-adjuvant. The mannan coating stabilized the bilosomes throughout the GI tract and also targeted the mannose receptors present on dendritic and macrophage cells. Oral vaccinations of mice with the modified bilosomes induced measurable cellular and humoral immune responses, as well as neutralizing sIgA which was considered as a key antibody produced during mucosal immune responses (Jain et al., 2005).

Glucomannosylated bilosomes containing BSA were found to induce mucosal, systemic as well as cell mediated immune responses (Jain et al., 2014). Oral vaccinations of mice with recombinant baculovirus displaying VP1 from human enterovirus 71 associated with bilosomes elicited significant VP1-specific humoral IgG and IgA immune responses (Premanand et al., 2013). In another study, diphtheria toxoid (DTx) loaded with nano-bilosomes was significantly immunogenic when administered orally in mice. Significant anti-DTx IgG and anti-DTx sIgA antibodies were detected in serum and mucosa of vaccinated mice, respectively (Shukla et al., 2011).

The optimized bilosome formulation was assessed as an oral vaccine delivery system with respect to its biodistribution and vaccine efficacy. The larger bilosome vesicles (∼6 vs. 2 µm in diameter) were demonstrated to have increased uptake within the Peyer’s patches. Oral immunization of ferrets with recombinant hemagglutinin (HA) incorporated into bilosome was able to reduce fever, suppressed lung inflammation and reduced viral load in the influenza challenge model. Thus, the bilosome has shown promising results as an oral vaccine carrier and could induce higher immune responses (Wilkhu et al., 2013).

Oral immunizations of mice using HA entrapped in bilosomes with particle sizes between 400–2000 nm elicited an immune response that was significantly biased towards Th1 as measured by serum antigen-specific IgG2a and splenocyte IFN-γ production rather than vaccination using bilosomes with size 10–100 nm (Mann et al., 2009). These results showed oral vaccine formulations could be physically modified to improve the effectiveness of vaccines to manipulate the required immune responses.

Oral immunization of bilosomes was able to induce both mucosal and systemic immune responses. Surface modification of bilosomes with anchoring ligands demonstrated their potential to target specific immune cells. Availability and low cost of bile salts and acids could thus transform such chiral carriers into attractive building blocks for targeting novel drug carrier systems. Moreover, bilosomes could enhance the bioavailability of drugs, increase the efficacy of drugs and the ability to entrap proteins, peptides and antigens (Palekar-Shanbhag et al., 2020).

## Immune-Stimulating Complexes

ISCOMs were first described by Morein et al. (1984) as a vaccine delivery vehicle (Bigaeva et al., 2016). ISCOMs are cage-like structures of 30–40 nm in diameter composed of glycosides that are present in cholesterol, Quil A, antigens and phospholipids (Fleck et al., 2019).

Oral vaccination with ISCOMs incorporating Herpes simplex virus type 2 (HSV-2) antigens (HSV-specific glycoproteins, gB2, gD1, gE1, and gG2) were able to induce high levels of IgA and IgG (systemic and local) as well as conferring sufficient protection against heterologous lethal dose of HSV-2 in mice (Mohamedi et al., 2001). In another study, ISCOMS containing a fusion protein comprising the OVA_323–339_ peptide epitope linked to CTA1-DD (a mucosal adjuvant) were highly immunogenic when administered by the subcutaneous (s.c.), oral, or nasal routes and induced a wide range of T cell-dependent immune responses as well as systemic immune responses (IgG2a and IgG1 isotypes). Their results demonstrated that ISCOMs could induce a broad range of cellular and humoral immunity (Mowat et al., 2001). Oral immunization of mice with two doses of influenza A/Sichuan/87 ISCOM vaccine were able to elicit robust humoral IgG2a subclass antibody and conferred protection against homologous virus challenge. The results demonstrated that ISCOM could be considered as a potential oral delivery system and provided adjuvanticity for viral antigens (Ghazi et al., 1995). Most of the available adjuvants in the market mainly activate the humoral immune response and there is a clear need for vaccines to induce a cellular immune response as well. ISCOM as an adjuvant could induce strong activation of both humoral and cellular immune responses and enhance the generation of most classes and sub-classes of antibodies.

## Metal-Based Nanoparticles

### Gold Nanoparticles

Gold (Au) has been used extensively in nano-medicine (in the form of NPs) due to its therapeutic effects on several diseases. Gold could also play an important role in the vaccine development field as a carrier and an adjuvant, enhancing the immunogenicity of antigens, reducing toxicity, and providing stability (Table 1) (Carabineiro, 2017).

The combination of delivery systems and adjuvants have been used to maximize the efficacy of mucosal vaccines. Chitosan functionalized gold NPs (CsAuNPs) were used as a carrier for tetanus toxoid (TT) as an antigen model along with the immunostimulant of Quillaja *Saponaria* extract (QS) as an adjuvant. Oral immunization of mice with CsAuNPs-TT-QS induced up to 28-fold immune responses (TT-specific IgG and IgA) compared to TT and TT-QS controls. Thus, combination of adjuvants with NPs can play an important role in the efficacy and stability of mucosal vaccines (Barhate et al., 2013).

The mucosal adjuvanticity of *Asparagus racemosus* extract (ARE) in oral delivery of TT using CsAuNPs as a carrier was evaluated in the murine model. A significant local and systemic increase in TT-specific IgG and IgA were observed when TT-CsAuNPs were formulated and delivered with ARE. As an immunomodulatory adjuvant for mucosal delivery of vaccines, ARE showed no effect on charge, size and loading properties of CsAuNPs. Additionally, ARE and CsAuNPs were observed to have no effects on antigenicity and the secondary structure of TT (Barhate et al., 2014).

### Silver Nanoparticles

Metallic NPs are relatively non-biodegradable, have rigid structures and are simple to synthesize. Currently, for green synthesis processing of metallic NPs, biological materials such as bacteria, plants and algae are usually used as capping groups and reducing agents. AgNP is one of the most vital and fascinating nanomaterials among several metallic NPs which have been exponentially used as antimicrobial and larvicidal agents because of the lower cost of production as well as the simplicity of synthesis. Recently, several studies have been conducted to use green synthesis NPs as adjuvants to increase the immunogenicity of antigens (Marslin et al., 2018).

AgNPs produced by the reduction of aqueous silver nitrate using leaf extract of *Eucalyptus* procera were evaluated to see if the immune response against inactivated rabies virus in the murine model was enhanced. The results were compared with commercially available alum adjuvant. The adjuvanting effects of green synthesized AgNPs on the potency of veterinary rabies vaccine were demonstrated with no *in vivo* toxicity (Asgary et al., 2016).

Jazayeri et al. (2012) synthesized green AgNPs by using PEG and β-D-glucose as stabilizer and reducing agents, respectively. Single oral immunization of one-day-old chicks with encapsulated H5 DNA vaccine with AgNPs rapidly increased production of antibodies against H5, cellular immune responses as well as enhanced cytokine productions. Moreover, PCR successfully detected the encapsulated H5 plasmid from the duodenum of the vaccinated chickens as early as 1 h post-immunization. Although there are some concerns about the toxicity of silver-based NPs, no toxicity induced by AgNPs as a carrier for oral DNA vaccine was observed in chickens (Jazayeri et al., 2012).

## Polymeric NPs

In recent years, synthetic polymer-based NPs/MPs have received more attention for their roles in vaccine delivery and adjuvanticity due to their ease in preparation, biocompatibility, biodegradability, stability in the biological environment, low cytotoxicity, protective, controlled and sustained-release of encapsulated substances (Calzoni et al., 2019).

### Poly(Lactic-co-Glycolic Acid)

The most commonly used synthetic biodegradable polymer-based NPs for vaccine delivery and adjuvanticity is PLGA. PLGA is a highly compatible copolymer of poly-lactic acid (PLA) and polyglycolic acid (Letchford and Burt, 2007). PLGA NPs can be easily loaded with a wide variety of molecules and have been approved for human and veterinary drug delivery by the American Food and Drug Administration (FDA) (Table 1) (Cappellano et al., 2019). PLGA based NPs could protect antigens in the harsh GI environment by surface attachment or encapsulation mechanisms using ionic or covalent bonding (Moon et al., 2012).

PLGA vaccine/adjuvant encapsulation could provide slow release of antigens and adjuvants which could stimulate innate immune responses and potentially induced both mucosal and systemic immune responses as well as improving humoral and long-term memory CD8 T cell (Kasturi et al., 2011; Demento et al., 2012). Besides encapsulation, the antigens could be adsorbed on the surface of PLGA by electrostatic or hydrophobic interactions (Pakulska et al., 2016).

Ashhurst et al. (2018) developed biodegradable PLGA as a carrier for the *M. tuberculosis* lipoprotein MPT83 together with the adjuvant trehalose-dibehenate (TDB) or Monophosphoryl lipid A (MPL). Mucosal immunization of mice with PLGA-encapsulated protein-based subunit vaccine was found to stimulate strong anti-MPT83 antibody or Th17 responses and PLGA could be considered as a potential carrier for vaccines against extracellular pathogens (Ashhurst et al., 2018).

PLGA-lipid NP hybrids were shown to be able to transfect plasmid DNA encoding a luciferase reporter gene into cells. The method of DNA loading to NPs, either absorbed on the outer surface or encapsulated within the NPs could affect the uptake of the NPs by the adherent and non-adherent cells, as well as the release of DNA (Zhong et al., 2010). Golan-Paz et al. (2018) developed a novel core-shell NP-based PLGA (core) and a multilamellar lipid shell when lipid bilayers are cross-linked between the two adjacent bilayers (PLGA-ICMVs). The PLGA-ICMV platform demonstrated great potential for encapsulating water-soluble biological agents such as protein and DNA plasmids and therefore could be considered as a promising candidate for therapeutic vaccine delivery (Golan-Paz et al., 2019).

The outer membrane protein W (OmpW) of Aeromonas hydrophila was cloned, purified, and encapsulated in PLGA NPs for oral vaccination of rohu (*Labeo* rohita Hamilton). The results showed oral administration of encapsulated OmpW using PLGA could provide dose-dependent protection against A. hydrophila infection in fish (Dubey et al., 2016).

To improve the efficacy of oral vaccine delivery, Taha-Abdelaziz et al. (2018) exploited PLGA for encapsulation and delivery of CpG to effector sites in the GI tract. Oral administration of PLGA-encapsulated oligodeoxynucleotides (ODN) containing unmethylated CpG and *C. jejuni* lysate enhanced the ability of CpG to reduce *Campylobacter* load in both layer and broiler chickens (Taha-Abdelaziz et al., 2018). In another trial by Alkie et al. (2017), encapsulated CpG with PLGA as a carrier showed higher and sustained innate immune responses in chicken macrophages and splenocytes compared to the naked soluble form of CpG (Alkie et al., 2017). The hydrophobic polymeric PLGA stabilized by one layer of phospholipids embedded in the surface area was used as oral vaccine delivery for Ovalbumin (OVA). Compared with the pure PLGA NPs, the lipid NPs achieved higher loading capacity and entrapment efficiency for the encapsulated OVA. Although the phospholipids were in a narrow size around PLGA, they showed all the applicable characteristics of both polymeric and liposome NPs, like preventing vaccine degradation, enhancing cellular absorption and low toxicity (Ma et al., 2014a).

Injured brain microvascular endothelial cells (BMECs) overexpressed tissue factor (TF) which was targeted by the fusion protein EGFP-EGF1 nanoparticles loaded with TF siRNA as a potential treatment. EGFP-EGF1 conjugated PLGA NPs (ENP) were used as a new targeted carrier for TF-specific siRNA which could be effectively delivered to atherosclerotic plaques *in vivo* and taken up by mouse vascular smooth muscle cells with high TF expressions *in vitro.* The data showed that the ENP-based transfections resulted in efficient downregulation of TF (Chen et al., 2013).

Ma et al. (2014) developed ulex europaeus agglutinin-1 (UEA-1) conjugated PLGA-lipid NPs containing a Toll-like receptor (TLR)-agonist monophosphoryl lipid A (MPL) as an oral vaccine delivery system. Oral immunization of mice with UEA-MPL/PLGA lipid NPs could protect and released the entrapped OVA through the GI tract. The entrapped ovalbumin (OVA) was protected from exposure to the GI tract and the OVA was released in a controlled manner. These results suggested that designed OVA-UEA-MPL/lipid NPs could be effectively transported by M cells, captured by mucosal dendritic cells (DCs) and induced mucosal IgA and serum IgG antibodies (Ma et al., 2014b).

### Poly (γ-glutamic Acid)

γ-PGA is a natural and promising biopolymer produced by several gram-positive bacteria such as *Bacillus* subtilis. Due to the unique characteristics of γ-PGA like low toxicity, biodegradability and biocompatibility with tissues and cells as well as non-immunogenic properties, it has been used extensively for vaccine development and pharmaceutical applications. γ-PGA NPs could induce cellular and humoral immune responses as well as having great potential as an antigen-delivery system and vaccine adjuvant (Uto et al., 2013).

Upon oral administration of γ-PGA in mice, the presence of γ-PGA was found in sub-epithelial dome region of Peyer’s patch (PP). Orally administered γ-PGA enhanced levels of various chemokines in intestines, accumulation of CD8 DC subsets in mesenteric lymph nodes and activation of DCs in Peyer’s patch. Oral inoculation of mixed γ-PGA with OVA was shown induce activation of OVA-specific T cells as well as levels of both IgA (intestinal) and IgG (sera). In the study, OVA was used as the model protein to develop a protein-based vaccine (Kim et al., 2019).

### Chitosan

Chitosan is a linear polysaccharide predominantly composed of β-(1–4)-linked D-glucosamine and N-acetyl-D-glucosamine. The N-acetyl-D-glucosamine moiety of chitosan is recognized by the mannose receptors on the DCs (Park and Babensee, 2012). The chitosan with amino and carboxyl groups interact with the glycoprotein in mucus to form a hydrogen bond which could produce an adhesive effect and absorption-enhancing properties for M cells of the follicle-associated epithelium (FAE) (Wang et al., 2011a). Due to chitosan mucoadhesion and transient opening of the tight junctions of the mucosal cell membrane, chitosan showed promising mucosal absorption effects. Interaction between the positive charge of chitosan and the negative charge of mucin could increase the contact time between the drug and the absorptive surface. Moreover, the mucoadhesion effect of chitosan was shown to increase the half-life of drug clearance (Ways et al., 2018).

Chitosan has attracted considerable attention as a biodegradable, non-toxic and biocompatible polymer to encapsulate a range of vaccines such as DNA, RNA, proteins, peptides as well as drugs as a novel delivery vehicle for both oral and intranasal administration. Various studies have shown chitosan as an oral vaccine delivery system by activation of macrophages, dendritic cells (DC) and lymphocytes which induce higher immune responses. Moreover, an ionic crosslinking method was used to encapsulate Newcastle disease virus (NDV) with chitosan NPs to enhance the efficacy of a lentogenic live-virus vaccine against ND. Single oral immunization of chickens with chitosan-live NDV provided full protection after challenge with a highly virulent NDV strain F48E9 (Zhao et al., 2012).

Recently, Renu et al. (2020) developed subunit chitosan NP based vaccine by using immunogenic outer membrane proteins (OMPs) and flagellin (F) protein (OMPs-F-CS NPs) of *Salmonella*. Oral immunization of layer chickens with OMP-F-CS NPs led to localization of the nanovaccine in ileal Peyer’s patches and induced significantly higher OMP-specific mucosal IgA production as well as lymphocyte proliferation response and reduced salmonellosis in poultry (Renu et al., 2020).

Chitosan NPs loaded with TOPO TA plasmid encoding Rho1-GTPase of *Schistosoma mansoni* were able to complex electrostatically with the plasmid and condensed it into positively charged nanostructures. The primary oral vaccination of mice with chitosan/Rho1-GTPase nanostructures followed by two boosters at two weekly intervals were demonstrated to induce high levels of IL-10. Furthermore, immunization of mice with only chitosan NPs conferred 47% of protection against parasite infection, suggesting an important role of chitosan in inducing a protective immune response against schistosomiasis (Oliveira et al., 2012). The protonated form of chitosan is normally water-soluble in acidic pH due to its pKa value of about 6. In order to improve the solubility and vaccine delivery, several studies have reported various chemical modifications of chitosan which improved stability, membrane permeability, mucoadhesivity and controlled release behaviour (Xing et al., 2018).

Alginate-coated chitosan microparticles could protect acid-labile drugs effectively from degradation in acidic pH than chitosan microparticles alone (Li et al., 2017) and enhanced antigen uptake by mucosal lymphoid tissues, especially at the Peyer`s patches (Zhang et al., 2016). Liu et al. (2013) showed alginate-coated chitosan NPs could be efficient and was observed to be safe carriers for the oral delivery of legumain DNA vaccines (Liu et al., 2013). In another study, Onuigbo et al. (2018) demonstrated oral administration of fowl typhoid vaccine encapsulated in alginate-coated chitosan microparticles could induce comparable innate and adaptive immune responses with the subcutaneous route of administration as well as protection from *S. gallinarum* virulent strain (Onuigbo et al., 2018).

The chitosan-alginate coated calcium phosphate NPs could protect the antigens in the GI environment against acidic degradation and enhance the immune response in the small intestine. The results demonstrated coating with chitosan enhanced antigen uptake by macrophages and Caco-2 (intestine epithelial cells) as well as improved surface expression of costimulatory molecules on macrophages. *In vivo* oral administration of alginate-chitosan-coated calcium phosphate-OVA NPs significantly enhanced the mucosal IgA and serum IgG antibody responses as compared to naked OVA, indicating that the chitosan- and alginate-coated calcium phosphate NPs could potentially be used as a promising oral vaccine delivery system (Cao et al., 2020).

In another study, bovine serum albumin (BSA) was used as a protein-based vaccine model and was loaded into the mannosylated chitosan NPs (MCS) by ionic gelation method with tripolyphosphate (TPP), followed by coating with Eudragit L100 (Eud) and electrostatic interaction. MCS NPs were accumulated more specifically into PPs after Eudragit L100 was dissolved in intestinal juices. Oral immunization of rats by using BSA-loaded Eudragit L100-coated MCS NPs elicited strong mucosal IgA and systemic IgG antibody responses. These results suggested that enteric-coated MCS NPs could serve as a promising carrier for oral protein-based vaccine delivery (Xu et al., 2018).

The effectiveness of ionotropic gelation method (by combining alginate and chitosan) as a bivalent streptococcus-lactococcus vaccine against *Streptococcus iniae* and *Lactococcus garvieae* was examined in rainbow trout. Oral vaccination of fish with chitosan-alginate coated microparticle vaccine increased immunity and improved the survival rate by the expressions of IL-6 and IgM (Halimi et al., 2019).

Oral immunization of turbots with carboxymethyl chitosan/chitosan NPs (CMCS/CS) loaded with extracellular products (ECPs) of *Vibrio anguillarum* showed elevated specific antibodies and higher concentrations of lysozyme and complement activities in fish serum than ECPs. CMCS/CS-NPs loaded with ECPs could improve both innate and adaptive immune responses and suggested that it could serve as a potential oral antigen delivery system in fish (Gao et al., 2016). The application of chitosan as a polycationic gene carrier for oral vaccine delivery has been ongoing since the 1990s. Chitosan NPs could protect DNA based vaccines against nuclease degradation by forming a polyelectrolyte complex with the negatively charged nucleotides and also improve the transfection efficiency. Kumar et al. (2008) used the porin gene of *Vibrio anguillarum* to construct a DNA vaccine by using pcDNA 3.1 expression vector. Oral vaccination of *Lates calcarifer* with chitosan encapsulated/plasmid complex showed antigen expression and moderate protection (46%) against *V. anguillarum* infection (Rajesh Kumar et al., 2008).

### Alginate

Alginate is a hydrophilic anionic polysaccharide obtained from the brown seaweed. Alginate can be orally administered or injected and has been extensively investigated in drug and vaccine delivery due to its low toxicity, biocompatibility, biodegradability (Lee and Mooney, 2012) and was approved by the U.S. Food and Drug Administration (Sosnik, 2014). As alginate is stable in simulated gastric fluid (pH 1.2), it was used to encapsulate an antigen to protect it from enzymatic degradation and facilitate its release (Borges et al., 2008).

Oral administration of rabbits and cattle with alginate microspheres containing *Pasteurella multocida* and OVA antigens could produce higher serum IgG, IgA, and sIgA responses than those immunized with unencapsulated antigens (Bowersock et al., 1999). In another study, oral immunization of mice with alginate encapsulated polysaccharide antigen of *Streptococcus pneumonia* showed effective protection against intranasal challenge with *S. pneumoniae* (Seong et al., 1999).

A formalin-killed *L. garvieae* TW-446.B3 was encapsulated in alginate microparticles and fish were orally immunized. The results demonstrated relative percent survival (RPS) of around 50% and this failed to warrant the efficacy of the vaccine formulation as a primary vaccination method. In another study, the efficacy of alginate-encapsulated killed vaccine as a booster immunization was evaluated 3 months after primary intraperitoneal immunization of fish with aqueous-based bacteria. The relative percent survival could reach up to 87%. These results highlighted the value of alginate encapsulation which led to an increase in the duration of protection of the rainbow trout against lactoccocosis (Romalde et al., 2004).

Moreover, Ballesteros et al. (2015) prepared alginate microparticles for oral delivery of the glycoprotein (G) gene of infectious hematopoietic necrosis virus (IHNV) to rainbow trout. The results demonstrated that the alginate microparticles could protect the DNA vaccine from degradation in the fish stomach and ensured early vaccine delivery to the hindgut, vaccine passage through the intestinal mucosa and its distribution through internal and external organs of vaccinated fish. Furthermore, single oral administration of encapsulated DNA vaccine in alginate microspheres induced dose-dependent adaptive immune responses and significant protection in rainbow trout (Ballesteros et al., 2015).

Bacille Calmette-Guerin (BCG) is the only approved vaccine against Tuberculosis. This vaccine is currently administered intradermally and has shown variable effectiveness of between 0 and 80% in various clinical trials (Colditz et al., 1995). As mycobacteria are generally transmitted through mucosal surfaces, the delivery of vaccines by mucosal routes can probably provide better immunity and protection. Intragastric gavage immunization of mice with BCG entrapped in alginate microspheres induced effective Th1 response in the spleen and provided effective protection against intravenous challenge 8 weeks after vaccination (Ajdary et al., 2007). Moreover, a single oral immunization of BALB/c mice with BCG encapsulated in alginate microspheres elicited effective mucosal as well as systemic immune responses in the lung and spleen (Hosseini et al., 2015). Alginate microspheres have been successfully used to encapsulate live porcine rotavirus or its recombinant VP6 protein as well as plasmid DNA for oral immunization of mice (Kim et al., 2002; Nograles et al., 2012). These investigations demonstrated alginate microspheres could be used as an effective oral carrier and adjuvant to induce effective mucosal and systemic specific immune responses. The muco-adhesiveness and muco-penetration of alginate increase the passage of the encapsulated drug through the epithelium, enhance the local and systemic drug delivery and increase the bioavailability and release of antigens.

Oral administration of encapsulated OVA (as a model antigen) in alginate microparticles to calves demonstrated that encapsulation of OVA in alginate microparticles could successfully induce pulmonary immunity and increased antigen-specific IgAs in bronchoalveolar lavage fluids (Bowersock et al., 1998). These studies showed that alginate microparticles were effective for encapsulation and oral administration of vaccines in small (fish, mouse, and rabbit) and large animals (cattle). However, it has yet to show potency for oral immunization in human clinical trials.

## Carbon NPs

In order to develop an effective oral vaccine carrier, immunological adjuvant, antigen protection and M cell antigen uptake, hydrophobic carbon NPs were synthesized by taking silica as a template and sucrose as a carbon source. Carbon NPs provided large mesopores and macropores which could encapsulate a large amount of antigens and could be considered as a potential antigen delivery system. Bovine serum albumin (BSA) was used as an antigen and loaded into the pores of carbon NPs. Oral immunization of mice with BSA/carbon NPs induced IgG in serum and mucosal IgA in salivary, intestinal and vaginal secretions as well as both T helper 1 and helper 2 mediated immune responses (Wang et al., 2011b).

## Conclusion

Oral vaccine development is complex and is considered the most challenging vaccination method due to the route of administration. Current licensed oral vaccines target mainly enteric pathogens and viruses that invade via the intestinal mucosa. In the last few years, material science applications in the field of vaccine development are growing rapidly and showed some promising results. Although the development of human oral vaccines have not been so successful, biocompatible and biodegradable NPs/MPs as vaccine carriers in animal models offer promising and novel vaccination methods that might act synergistically both as a delivery vehicle and an adjuvant. Engineered NPs/MPs have demonstrated their potentials to ensure the induction of both cellular and humoral immune responses as well as offering much greater advances for the future development of oral vaccines for humans. The progress of developing oral vaccines required careful consideration of multiple physicochemical and biological barriers in the GI tract as well as delivery systems and adjuvants. However, with increased understanding of intestinal biology, mucosal immunity and the next generation of NPs/MPs as vaccine carriers and adjuvants, there is great hope to address the limitations and develop more novel oral vaccines.
